# A xylan assisted surface-enhanced Raman scattering substrate for rapid food safety detection

**DOI:** 10.3389/fbioe.2022.1031152

**Published:** 2022-09-30

**Authors:** Zhouyang Xiang, Mengyun He, Li Li, Jamshed Bobokalonov, Abduvali Dzhonmurodov, Xingxiang Ji

**Affiliations:** ^1^ State Key Laboratory of Biobased Material and Green Papermaking, Qilu University of Technology, Shandong Academy of Sciences, Jinan, China; ^2^ State Key Laboratory of Pulp and Paper Engineering, South China University of Technology, Guangzhou, China; ^3^ V.I. Nikitin Institute of Chemistry, National Academy of Sciences of Tajikistan, Dushanbe, Tajikistan

**Keywords:** xylan, bimetal nanoparticle, paper base, surface-enhanced Raman scattering, food safety detection

## Abstract

Cellulose fiber/paper-based surface-enhanced Raman scattering (SERS) is considered as a promising food safety detection technology due to its non-toxicity, low cost, flexibility, and hygroscopicity for possible rapid on-site agricultural product contaminant detection. However, it faces the problems of poor noble metal adhesion and toxic noble metal reducing agent. In this study, a natural macromolecule—xylan was used as both a reducing agent and a stabilizing agent to prepare stable Au-Ag bimetal nanoparticles, which were anchored on the paper surface by xylans in order to fabricate a paper-based Au-Ag bimetallic SERS substrate. The results show that the SERS substrate has a high Raman enhancement performance and reproductively. The substrate can effectively detect trace pesticide, i.e., thiram, and the limit of detection is as low as 1 × 10^–6^ mol/L (0.24 ppm). In addition, the paper-based SERS substrate can be used for direct detection of pesticide residues on the surface of fruit. The paper-based SERS substrate developed in this study has great potential in applications for rapid food safety detection.

## 1 Introduction

Since food safety has become a worldwide issue, in the process of food production, processing, preservation, packaging, transportation and consumption, monitoring contaminants such as pesticides, antibiotics, illegal additives, bacteria, pathogens and heavy metals in food is of great significance (Hu et al., 2021a). Currently, the commonly used detection methods include Enzyme Linked Immunosorbent Assay (ELISA), High Performance Liquid Chromatography (HPLC), Gas Chromatography-Mass Chromatography (GC-MS), High Performance Thin-layer Chromatography, Supercritical Fluid Extraction, etc. (Guo et al., 2021). These methods are labor-intense, time-consuming and require expensive instruments and skilled workers, which are difficult to meet the requirements of rapid on-site detection and screening a large batch of samples. Therefore, it is urgent to develop sensitive, efficient and convenient food detection technology.

Surface-enhanced Raman scattering (SERS) is considered as a promising food safety detection technology due to its nondestructive, fingerprint and high sensitivity. In recent years, with the development of nanotechnology, researchers have established a series of rapid monitoring methods for common food contaminants (mycotoxins, drug residues, pesticides and illegal additives, etc.) based on SERS technology. Generally, traditional SERS active substrates are generally composed of noble metals (such as Au, Ag, Pt, etc.) in the form of nanoparticle aggregation or array (Hu et al., 2021a). Localized surface plasmon resonance (LSPR) excited on the surface of metal nanoparticles (NPs) generates a strong electromagnetic field to achieve the enhancement of Raman signals. The dominant enhancement often observed is the electromagnetic enhancement of targeted molecules adsorbed on the metal surface, with the enhancement factors ranging from 10^4^ to 10^12^ (Zong et al., 2018). Non-metallic materials such as semiconductor, graphene quantum dots and photonic crystal fiber have also been used to prepare SERS substrate due to their high chemical stability, good biocompatibility and high carrier mobility (Yang et al., 2019; Beffara et al., 2020). However, rigid solid SERS substrates such as metals, silicon wafer, glass or graphite are expensive and not suitable for direct *in-situ* detection of irregular-shaped agricultural products, which hinders the practical application of SERS technology. In recent years, due to their weak SERS response, low background signal and interference signal during detection, cellulose fibers or paper bases are used as the supports for noble metals to prepare flexible SERS substrates, which are considered to be one of the most promising materials for preparing test paper for *in-situ* food detection.

Recent developments of using various forms of cellulose, such as cellulose fiber, cellulose membrane, paper base, cellulose nano-fiber (CNF), cellulose nano-crystal (CNC), bacterial cellulose, and various cellulose derivatives in fabricating functional materials have been extensively studied (Ling et al., 2022a; Ling et al., 2022b). The applications of different forms of cellulose in SERS substrate for food safety detection have been explored. Vávrová et al. (Vávrová et al., 2022) successfully prepared Au NPs using dicarboxylic cellulose (DCC) and hyaluronate (DCH) as reducing agent and end-capping reagent, and applied the Au NPs to detect the Raman signal of N-acetylcysteine (NAC). The enhanced effect was subjected to further improvement, which may be due to the presence of DCC and DCH hinder the contact of NAC with Au NPs. Marques et al. (2008) used sodium citrate as a reducing agent to prepare Ag NPs/BC composite by *in-situ* reduction, and used it as a surface‐enhanced Raman scattering (SERS) substrate to detect thiosalicylic acid and 2,2-dithio-bipyridine. The detection limit was about 10^−4^ mol/L, but there is room for improvement in sensitivity. Barbosa et al. (2021) prepared a novel SERS substrate by coating a layer of Ag NPs on the β-D-glucan film by laser ablation method. The SERS activity was evaluated by using crystal violet as probe molecules. The results showed that the enhanced effect reached 10^6^, which means that this substrate had stronger SERS effect than silver colloid. Hu et al. (2021b) successfully prepared a 3D cellulose nanocomposite doped with Au NPs by one-pot method, and the Raman signal of R6G can be collected on its surface at a concentration of 1 × 10^–9^ mol/L, which indicates that the sensitivity of this SERS substrate is greatly improved. In addition, due to the porous structure of the SERS substrate, melamine in milk could be directly detected with a detection limit of 2.5 mg/kg. Significantly, a good SERS base should ensure homogeneity stability and repeatability in trace detection. Therefore, it is necessary to utilize chemical reducing agents and stabilizers to control the size and shape of metal nanoparticles during the preparation of SERS substrates. Due to the weak reducing ability of cellulose, compounds such as NaBH_4_, hydroxylamine, CTAB, and PVP were often added as reducing agents or stabilizers. On the one hand, such compounds are toxic and unfriendly to the environment; on the other hand, the reducing agents and stabilizers doped in the substrate may interfere with the SERS signal.

Polysaccharides are the most abundant natural polymers and widely found in animals, plants and microorganisms. Common polysaccharides include hemicelluloses, pectin, starch, chitosan, etc. The differences in sugar compositions and molecular chain structures endow polysaccharides with unique physicochemical properties. In recent years, the use of polysaccharides and their derivatives as reducing agents or stabilizers to prepare metal nanoparticle for SERS substrate materials has aroused the interests of researchers (Zhao et al., 2015; Puente et al., 2021). Xylan is a type of hemicelluloses, widely present in various lignocellulosic biomass. Due to the containing of abundant reducing functional groups, e.g. hydroxyls and aldehydes, and its unique rheological properties, it can be used as a green reducing agent and stabilizer for the preparation of metal nanoparticles (Zong et al., 2018). Compared with other reducing agents, such as NaBH_4_ and hydrazine hydrate, xylan is more environmentally friendly and does not require additional stabilizers. Using xylan as the reducing agent and stabilizer, Cai et al. (Cai et al., 2019) prepared two kinds of nanoparticles, Au@Ag and Au-Ag, with different shell thicknesses by microwave heating-assisted reduction and applied them to fabricate SERS substrate; SERS substrate with embedded xylan wrapped Au@Ag particles had an excellent enhancement effect on Raman signal of 4-mercaptobenzoic acid and a detection limit as low as 1 nmol/L. Bi-metal core-shell structure is more advantageous than single metal SERS substrate materials (Guo et al., 2019). Firstly, the surface plasmon resonance effect could be regulated by adjusting core-shell composition ratio. Secondly, the protective effect of the shell can improve the stability of the materials. Although core-shell structure has an excellent SERS effect, its application process involves multiple steps, i.e. mixing the analyte with the core-shell particles before detection, which could cause agglomeration.

Aiming at the above problems, an environmental friendly and flexible SERS substrate was designed. Xylans were used as a green reducing agent and stabilizer, while chloroauric acid was used as Au precursor to prepare Au NPs, which were immobilized on paper base by coating. Tollens reagent was then used as the Ag precursor and microwave-assisted reduction was used to prepare SERS substrates loaded with Au-Ag bimetallic NPs. The SERS activity and signal uniformity of the substrate were evaluated by using thiram as the probe molecule. Finally, the *in-situ* detection of pesticide residues on real fruit surfaces using this paper-based SERS substrate was conducted.

## 2 Experimental

### 2.1 Material

Sodium chlorite, gold (III) chloride trihydrate, ammonium hydroxide solution, tetramethylthiuram disulfide (thiram) were purchased from Shanghai Macklin Biochemical Co., LTD. (Shanghai, China). Silver nitrate was obtained from Shanghai Fine Chemical Materials Research Institute (Shanghai, China). Sugarcane bagasse used in the experiment was supplied by Xinping Nan’en Sugar and Paper Manufacturing Co., Ltd. (Yunnan, China). The filter paper was purchased from General Electric Biotechnology Co., Ltd. (Hangzhou, China).

### 2.2 Extraction of xylan

The oven dried bagasse raw materials was first dewaxed in a Soxhlet apparatus with 95% ethanol for 6 h. After dewaxing, bagasse was successively bleached with 4 wt% NaClO_2_ solution and 2 wt% NaClO_2_ solution (w/w) at 75°C and pH 3.9–4.0 for 1 h. The solid-liquid ratio was 1:12 (w/v). The obtained solid residue, i.e., holocellulose, was washed three times with deionized (DI) water and one time with anhydrous ethanol. The holocellulose was extracted with 2 wt% NaOH at 90°C for 3 h under stirring with a solid/liquid ratio of 1:20 (w/v, g/mL). After the filtrate was neutralized to pH 5.5 with acetic acid, the filtrate was concentrated to 1/3 of its original volume by rotary evaporation and then was precipitated in 95% ethanol of the volumes of three times of the concentrated filtrate. Finally, the xylan was separated by centrifugation, washed with 75% ethanol and freeze-dried for further use. The compositional analysis of xylan followed the methods described by Jin (Jin et al., 2019).

### 2.3 Preparation of the paper-based Au-Ag bimetallic SERS substrate

The extracted xylan was used as reducing agent and stabilizer to prepare gold nanoparticles (Au NPs) according to the method reported by Luo (Luo et al., 2015). In detail, 0.12 g of xylan was dispersed in 3 ml DI water, and HAuCl_4_ 3H_2_O of different mass (5–40 mg) was dissolved in 12 ml DI water. The two were mixed. The reaction temperature and time varied between 60–100°C and 10–80 min, respectively. The sample was properly diluted and the characteristic adsorption peak of Au NPs were detected by UV spectrophotometer with a scanning range of 300–800 nm.

The xylan/gold nanoparticle dispersion prepared by the reaction of 0.12 g xylan and 5–40 mg HAuCl_4_ 3H_2_O at 80°C for 20 min was coated on the surface of the paper base by a hand-coating machine. Filter paper sheets were cut into 3 cm × 3 cm specimens. Each specimen was coated both sides and each side was coated 10 times to control the amount of Au NPs. In each time of coating, 0.15 mL of the xylan/gold nanoparticle dispersion was used. After each time of coating, the paper base was placed in an oven at 60°C to dry for 15 min, and then the second coating was performed. The gold nanoparticles-filter paper (Au-FP) was finally obtained.

According to the method of Luo (Luo et al., 2015), Au-Ag bimetallic nanoparticles were prepared. In detail, the Au-FP was placed in a three-port round-bottom flask with xylans and Tollens reagent added and the mixture was subjected to 30-min microwave reaction at 800 W and 65°C. Tollens reagents were prepared by 0.1 mol/L AgNO_3_ solution, 2 wt% NaOH solution and 2 wt% ammonium hydroxide; the amount of 0.1 mol/L AgNO_3_ used was varied to give different Ag/Au mole ratio ranging from 0.5 to 10 (the mole of Au was calculated based on the amount of xylan/gold nanoparticle dispersion used for coating). They were then dried in a vacuum oven for 24 h to obtain different Au-Ag-FP. Different Au-Ag bimetallic nanoparticles are denoted as Au-Ag_m_-n, where m represents Ag/Au mole ratio (m varied from 0.5 to 10), n represents different amounts of xylan added when preparing Ag (n = 0, 1, 2, 3, 4, 5, and 6 correspond to 0, 5.71 × 10^–8^, 5.71 × 10^–7^, 5.71 × 10^–6^, 5.71 × 10^–5^ and 5.71 × 10^–4^ mol of anhydrous xylose units). The surface morphology and elements distribution were characterized by FESEM (LEO1530VP, Carl Zeiss, Germany). The electron valence states and chemical properties of Au and Ag were determined by X-ray photoelectron spectroscopy (XPS) (K-Alpha, Thermo Fisher, United States). Au-Ag bimetallic nanoparticles embedded on the paper surface were separated by ultrasound and centrifugation. The nanoparticles were then evaluated by a UV spectrophotometry with a scanning range of 300–800 nm and a transmission electron microscopy (Talos F200s, Thermo Scientific, United States).

### 2.4 Analysis of the SERS performance of substrates

To evaluate the Raman enhancement performance of different Au-Ag-FP, 10 μL thiram (concentration between 1 × 10^−3^–10^–6^ mol/L) solution was dropped onto the substrate and placed in a 50°C oven for 5 min. After ethanol volatilization, a Micro-Raman Spectroscopy System (LabRAM Aramis, Horiba Jobin Yvon, France) with excitation wavelength of 633 nm was used to collect the spectra of the substrate. In addition, on the same Au-Ag_8_-3-FP substrate, after dropping thiram solution, 20 points were randomly selected for SERS detection.

In order to test the feasibility of *in-situ* extraction and detection of pesticide residues on fruit surface with the substrate, slightly wetted Au-Ag_8_-3-FP substrate was used to wipe the surface of apple containing thiram, and then the Micro-Raman Spectroscopy System was used to irradiated the substrate to collect SERS spectra.

## 3 Results and discussion

### 3.1 Characterization of Au nanoparticles

The extracted sugarcane bagasse xylan had a sugar composition of 58.6% xylose, 17.2% glucose, 9.1% arabinose, 2.1% galactose, 1.2% mannose and 5.3% uronic acid. Previous studies have found that the presence of side-chain groups plays an important role in preventing the crystallization/aggregation of xylan molecules, and the low-branching xylan extracted from bagasse is easy to crystallize and cannot form water casting film (Xiang et al., 2020). Based on previous studies, the branching degree of xylan used in this study (arabinoglycan + uronic acid/xylan) was deliberately controlled at about 0.24, thus providing good film formation and suitable for coating applications in this paper.

**FIGURE 1:**
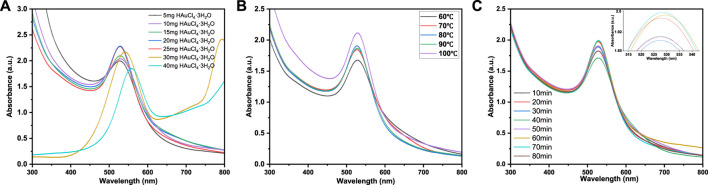
UV-vis spectra of xylan/Au NPs dispersions at **(A)** different reactant ratios (0.12 g Xylan: 5–40 mg HAuCl_4_·3H_2_O), **(B)** different reaction temperature and **(C)** different reaction time.

Au NPs prepared by xylan as reducing agent were characterized by UV-vis spectroscopy. In Figure 1, a strong adsorption peak can be observed at 524 nm, which is the characteristic surface plasmon resonance peak (SPR) of Au NPs, indicating the formation of Au NPs (Chen et al., 2012). In Figure 1A, when 5 mg HAuCl_4_·3H_2_O was used as gold source, the adsorption peak was weak, indicating that very few Au NPs were formed under this condition. With the increase of Au^3+^ concentration, a strong and narrow peak appeared in the UV spectra, indicating that the amount of Au NPs increased. When the amount of HAuCl_4_·3H_2_O increased to 25 mg, the adsorption peak was the largest among different amounts of HAuCl_4_·3H_2_O addition. As the amount of HAuCl_4_·3H_2_O further increased, the adsorption peak became weaker and wider with red shift. This is due to Au NPs aggregation, resulting in the weak adsorption strength of Au NPs at 524 nm. Furthermore, the larger size of Au NPs may lead to longitudinal plasma resonance due to plasma coupling between particles (Sun et al., 2004). The absorbance of Au NPs coupled plasma in close contact leads to red shift in UV-vis spectra, and SPR broadening indicates that the surface ligand provides a new relaxation channel for plasma excitation (Itoh et al., 2004). All these phenomena indicate that excessive HAuCl_4_·3H_2_O lead to aggregation of Au NPs. Therefore, the suitable mass ratio is 0.12 g xylan: 25 mg HAuCl_4_·3H_2_O, which was selected in subsequent experiments.

Figure 1B shows the UV-vis spectra of Au NPs prepared at different reaction temperatures. At 60°C, the absorption peak intensity was low because the reaction was incomplete due to the low temperature, and fewer Au NPs were generated. When the reaction temperature was raised to 80°C, the absorbance value of UV-vis spectra reached the maximum, but when the reaction temperature was raised further, the absorption peak widened. This may be due to the relatively high temperature that the generation of a large amount of Au NPs may lead to the agglomeration phenomenon of AuNPs, thus widening the SPR absorption peak and redshifting the absorption peak. Therefore, 80 °C was chosen as the optimum reaction temperature for subsequent experiments.

In Figure 1C, the influence of reaction time on Au NPs formation was discussed. With the increase of reaction time from 10 min to 20 min, the intensity of SPR peak increases significantly, confirming the formation of Au NPs. It should be noted that at 20 min, the SPR absorption peak intensity of xylan/Au NPs composite already shows a relatively high value. Further increase of heating time, the SPR absorption peak intensity does not vary significantly. At 80 min, the peak intensity even decreases significantly, indicating that long reaction time leads to Au NPs aggregation and unstable xylan/Au NPs composite. Therefore, the optimal reaction time is determined to be 20 min.

**FIGURE 2:**
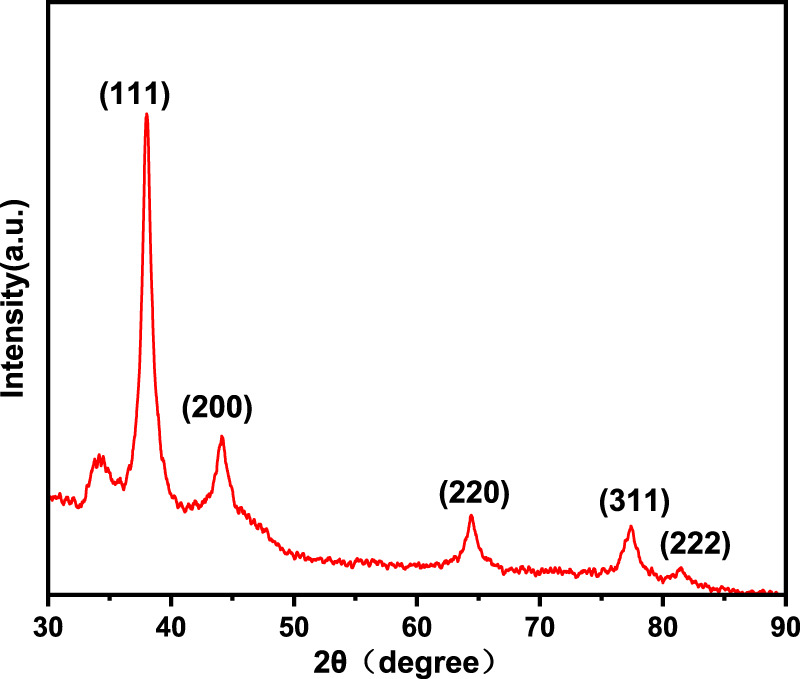
X-ray diffraction patterns of Au-FP.

The XRD spectrum of Au-FP is shown in Figure 2. The diffraction peaks of Au NPs are at 2θ = 38.02°, 44.11°, 64.45°, 77.42°, 81.49° correspond to the (111), (200), (220), (311), and (222) planes of Au, respectively. These peaks match well with the diffraction peaks of standard Au NPs (JCPDS No. 04–0784) and is consistent with previous literatures (Luo et al., 2015).

### 3.2 Characterization of the paper-based Au-Ag bimetallic SERS substrate

**FIGURE 3:**
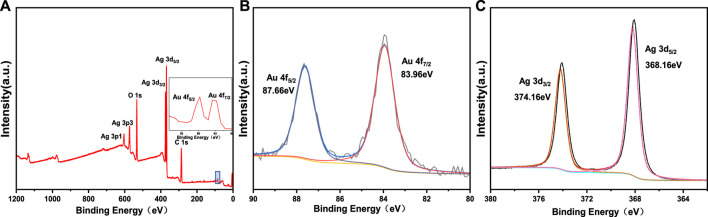
XPS spectra of Au-Ag_8_-3-FP: **(A)** wide scan spectra, **(B)** high-resolution XPS sprectra of Au 4f, **(C)** high-resolution XPS sprectra of Ag 3 d

In order to determine the chemical valence state of Au and Ag, Au-Ag-FP was characterized by XPS, and the results were calibrated with C1s = 284.8eV binding energy standard, as shown in Figure 3. In Figure 3A, Au and Ag are found on the surface of Au-Ag-FP in addition to C and O. The C and O may be derived from xylan or cellulose on the surface of paper base, while the peaks at Au 4f and Ag 3 days are derived from Au-Ag bimetal nanoparticles on the paper base.

In the XPS narrow spectrum scanning of Au 4f (Figure 3B), the peaks with binding energies of 87.66 eV (4f5/2) and 83.96 eV (4f7/2) correspond to Au (0). In the XPS narrow spectrum scanning of Ag 3 days (Figure 3C), the peaks of 374.16 eV (3d3/2) and 368.16 eV (3d5/2) are the characteristic peaks of Ag (0). It shows that xylan has good reducibility so that Au^3+^ and Ag^+^ are successfully reduced to Au and Ag.

**FIGURE 4:**
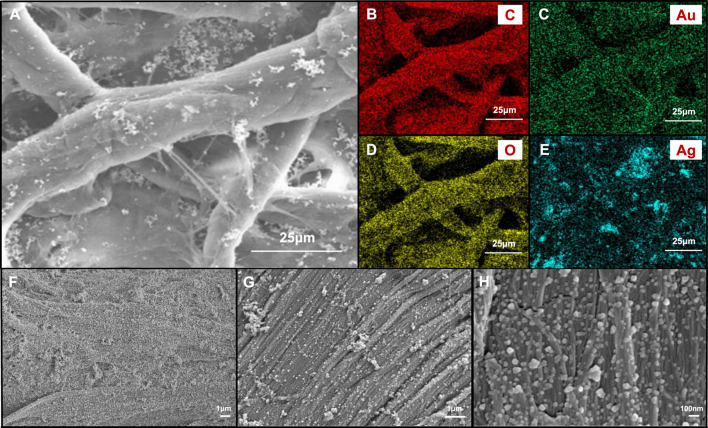
**(A)** FESEM images of Au-Ag_8_-3-FP surface and images with elemental mapping of **(B)** C, **(C) **Au, **(D)** O, **(E)** Ag, respectively. FESEM images of Au-Ag_8_-3-FP surface with **(F)** 5 k magnification, **(G)** 10 k magnification and **(H)** 50 k magnification.

**FIGURE 5:**
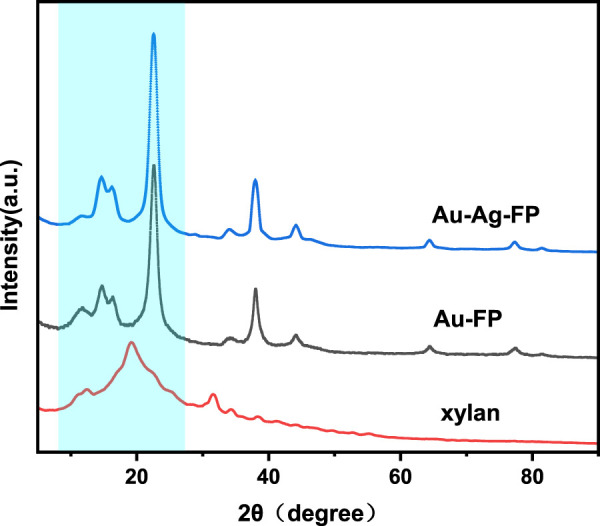
X-ray diffraction patterns of Au-Ag-FP, Au-FP and xylan.

In order to further understand the dispersion of Au-Ag on the paper surface, the surface morphology and surface element distribution of Au-Ag_8_-3-FP surface were characterized by FESEM (Figure 4). From Figure 4H, it can be observed that a large number of metal nanoparticles are attached to the surface of the paper base, and the distribution is relatively uniform, with only a slight aggregation phenomenon. However, when the magnification is reduced to 10 k and 5 k, some agglomeration occurs. Through the element distribution map, it can also be found that some aggregations of Ag NPs can be observed. It indicates that although the hydroxyl groups on xylan can coordinate and interact with metal nanoparticles, the ability to stabilize metal nanoparticles is not ideal; when the concentration of metal nanoparticles is high, aggregation is easy to occur. In Figure 5, it can be observed that there are some obvious XRD diffraction peaks between 10–35°. These diffraction peaks belong to the xylan hydrate crystals. Since alkali extraction cleaves the acetyls on xylan, xylans with low degree of branching may crystalize with water forming xylan hydrate crystals (Xiang et al., 2020). The xylan extracted from sugarcane has a high degree of branching, and its XRD pattern shows a mostly amorphous structure. However, the crystallinity of the xylan after the reaction increases significantly with the appearance of evident characteristic XRD peaks of xylan hydrate crystals (Li and Xiang, 2022). The forming of xylan hydrate crystals may be due to the destruction of xylan branches during the reaction with Tollens reagent at high pH (Xiang et al., 2020).

**FIGURE 6:**
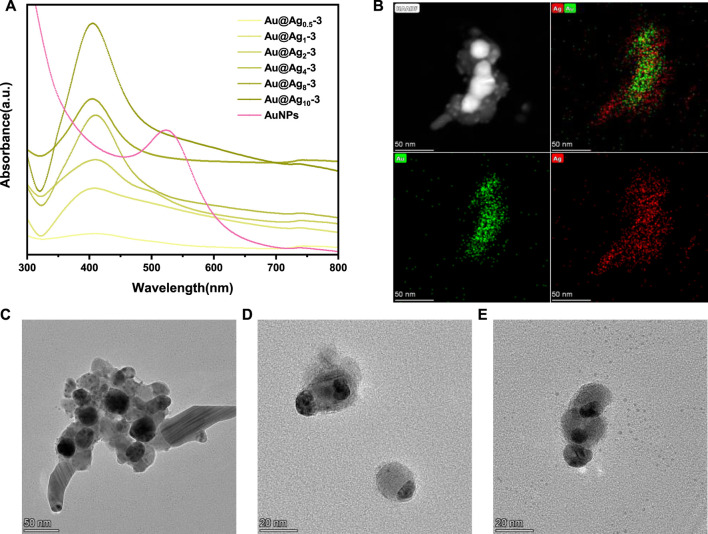
**(A)** UV-vis spectra of the nanoparticles on Au-Ag_m_-3-FP, **(B)** STEM dark-field picture and EDS mapping pictures of nanoparticles on Au-Ag_8_-3-FP, **(C)**, **(D)**, **(E)** TEM images of nanoparticles on Au-Ag_8_-3-FP.

As mentioned above, Au NPs exhibit a strong surface plasmon resonance peak at 524 nm. According to previous studies, the adsorption peak of silver nanoparticles is generally at around 410 nm, and red shift or blue shift would occur with the change of particle size (Zhang et al., 2007). In Figure 6A, it can be found that with the increase of silver content, the resonance of silver gradually dominates and the adsorption peak gradually blue shifts. In order to further explore the structure of nanoparticles, STEM dark-field images and EDS elemental mapping tests were carried out. As can be seen from Figure 6B, multiple nanoparticles with a bright inner core and a dark outer shell aggregate together, with gold element in the inner core and silver element in the outer shell, suggesting the successful formation of bimetallic core–shell nanoparticles (Cai et al., 2019). Figures 6D,E shows that some Ag NPs do not completely wrap the Au NPs, which may be due to that, after the Au NPs are coated on the paper-based surface, only the exposed surface can be in contact with the Ag NPs. Figures 6C–E show that some Au-Ag nanoparticles have slight agglomerations, which may be caused by the ultrasonic and centrifugal operation when Au-Ag bimetal nanoparticles are separated from the paper-based surface.

### 3.3 The SERS performance of the paper-based Au-Ag bimetallic SERS substrate

**FIGURE 7:**
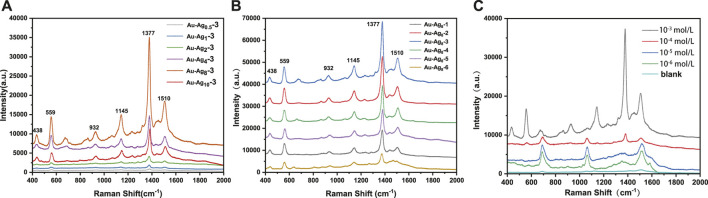
**(A)** The Raman spectra of thiram at 10^–3^ mol/L on the surface of Au-Ag_m_-3-FP, **(B)** the Raman spectra of thiram at 10^–3^ mol/L on the surface of Au-Ag_8_-n-FP, **(C)** the Raman spectra of different concentrations (10^−3^–10^–6^ mol/L) of thiram on the surface of Au-Ag_8_-3-FP.

The Raman enhancement performance of Au-Ag-FP substrates prepared with different Au/Ag molar ratios was evaluated, and the results were shown in Figure 7A. After thiram was dropped on the surface of Au-Ag_m_-3-FP, the strongest Raman spectrum peak of 10^–3^ mol/L thiram was located at 1377 cm^−1^, corresponding to the stretching vibration of C-N bonds and the symmetric stretching vibration of CH_3_. The peak at 1145 cm^−1^ is attributed to the antisymmetric vibration of CH_3_, the peak at 1510 cm^−1^ and 1145 cm^−1^ corresponds to the stretching vibration of C-N and the in-plane bending vibration of CH_3_, the stretching vibration peak of C=S and CH_3_N corresponds to the peak at 932 cm^−1^, and the vibration peak of S-S is at 559 cm^−1^. The peak at 444 cm^−1^ corresponds to the bending vibration of CH_3_NC and the stretching vibration of C=S (Kang et al., 2002). With the increase of Ag dosage, the Raman enhancement effect of the substrate is obviously enhanced. When the mole quantity of Ag is 8 times of Au (Au-Ag_8_-3-FP), the Raman intensity is the highest, which is about 80 times of Au-Ag_0.5_-3-FP. However, with further increase of Ag dosage, the enhancement performance of the substrate decreases, which may be due to the stabilizing effect of xylan on nanoparticles is not strong enough, leading to agglomeration phenomenon (Figure 4H) and uneven size and shape of Au-Ag particles.

Raman enhancement performance of Au-Ag_8_-n-FP prepared with different amounts of xylan is shown in Figure 7B. With the increase of xylan addition during Ag NP preparation, the Raman spectra of 10^–3^ mol/L thrima dropped on Au-Ag8-n-FP have increased intensities for peaks at 559, 932, 1145, 1377 and 1510 cm^−1^. Au-Ag_8_-2-FP and Au-Ag_8_-3-FP have the largest intensity, which indicates that they have a strong Raman enhancement. However, with further increase of the xylan addition during Ag NP preparation, the peak intensities of the substrates (Au-Ag_8_-4-FP, Au-Ag_8_-5-FP and Au-Ag_8_-6-FP) decreases. This phenomenon indicates that the addition of xylan is beneficial to reducing the aggregation of nanoparticles, leading to a strong Raman enhancement effect. However, it can also be postulated that with further increase of the xylan dosage, excessive xylans may cover or bury the Ag NPs leading to a weakened Raman enhancement effect.

In order to explore the practical application performance of the SERS substrates, the Raman enhancement performance of Au-Ag_8_-3-FP on common pesticide pollutant thiram was evaluated. As can be seen from Figure 7C, the higher the thiram concentration, the stronger the SERS signal peak is. In the SERS spectrum for 10^–3^ mol/L thiram, very strong signal peaks are observed at 1377 cm^−1^, 1510 cm^−1^, 1145 cm^−1^ and 559 cm^−1^. With the decrease of thiram concentration, the signal peak intensity at 1377 cm^−1^ weakens significantly, but the signal peak at 1510 cm^−1^ increases. This may be due to that at a low concentration, the thiram adsorbed on the Au-Ag may not completely cover the metal surface. The charges transferred from thiram molecule to metal redistribute the electron density of thiram and enhance the intensity of Raman peaks (Dao et al., 2021).

**FIGURE 8:**
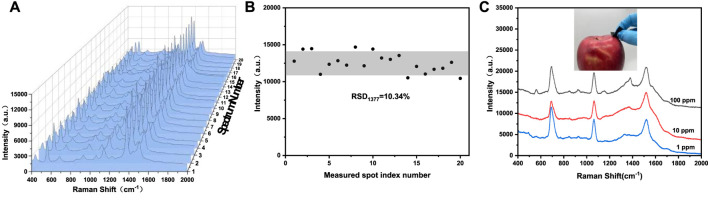
**(A)** Raman spectra of 200 ppm thiram collected from 20 randomly selected spots from the same Au-Ag_8_-3-FP substrate, **(B)** the scatter diagram of intensity distribution of the 1377 cm^−1^ peak of thiram corresponding to spectra in **(A)**, **(C)** Raman spectra of thiram with different concentration swabbed from the apple surface.

Practical application of SERS substrate not only requires high sensitivity, but also requires good reproducibility. The reproducibility of Au-Ag_8_-3-FP substrate was studied by using thiram as the probe molecule. Approximately 15 μL 200 ppm thiram ethanol solution was dropped on the substrate, and then 20 points were randomly selected to collect the SERS spectrum of thiram. The SERS spectra of different points (Figure 8A) show high similarity in quantity, location and intensity of characteristic peaks. Figure 8B plots the intensity of Raman characteristic peak at 1377 cm^−1^ from the 20 points, which is used to demonstrate the reproducibility of SERS base. According to the standard formula of relative intensity deviation of SERS spectrum (Luo et al., 2015), RSD was calculated to be 10.43%, showing good reproducibility. The paper-based SERS has good flexibility, which enables the substrate to make good contact with the surface of the object to be measured. The three-dimensional porous structure of filter paper acts as a capillary, giving it good water adsorption performance. Based on the above characteristics, the use of Au-Ag_8_-3-FP substrate should be able to achieve effective extraction and rapid detection of complex sample surface. The Au-Ag_8_-3-FP substrate was slightly wetted with 15 μL ethanol solution, and then the apple surface was dried after dropping with 15 μL thiram ethanol solution. The SERS spectrum of the substrate collected with thiram was shown in Figure 8C. The characteristic peak of thiram could still be observed when the solution concentration is as low as 1 ppm. The results show that SERS substrate could be used for rapid extraction and detection of pesticide residues on real vegetable and fruit surface.

In recent years, it has been reported that various metal nanoparticles supported by cellulose as the substrate were used for food safety SERS detection (Table 1). The SERS substrate developed in this study has better or comparable performance in detection limits compared to some recent studies (Table 1). In addition, the detection limit of Au-Ag_8-3_-FP in this study is 1 × 10^–6^ mol/L (i.e. 0.24 ppm), which is lower than the maximum thiram residue level of 7 ppm regulated by US Environmental Protection Agency (Sun et al., 2017) and 5 ppm required by the National Food Safety Standard of China (Zhang et al., 2019) indicating that the substrate developed in this study can potentially be used in food safety detection.

**TABLE 1:** Cellulose-based SERS material for food safety detection.

Cellulose form	Substrate	Analyte	Method	LOD^a^	References
filter paper	Au-Ag/filter paper	thiram	*in-situ* reduction	0.24 ppm	This paper
	Ag@SiO_2_/filter paper		Droping	1 nmol/L	Sun et al. (2019)
	AgNPs/AKD/filter paper		Droping	0.46 nmol/L	Lee et al. (2018)
	AgNPs/filter paper		Droping	1 ppm	Zhang et al. (2019)
	Au@Ag cellulose membrane/filter paper		transfer	0.24 ppm	Lin et al. (2019)
cellulose	Ag coated cellulose		magnetron sputtering	0.20 ppm	Gao et al. (2020)

a LOD, limit of detection.

## 4 Conclusion

Using natural macromolecule xylan as a reducing agent, a stabilizing agent and an anchoring agent, Au-Ag bimetal nanoparticles were successfully prepared and steadily anchored on the paper base to fabricate a paper-based Au-Ag bimetallic SERS substrate. Different SERS substrates were prepared by adjusting the amounts of xylan, HAuCl_4_·3H_2_O and Tollens reagent. The results show that these SERS substrates have high Raman enhancement performance and reproductively. The substrate fabricated through Ag/Au mole ratio of 8 and xylan addition during Ag preparation of 5.71 × 10^–6^ mol has the highest Raman intensity. The substrate can effectively detect trace pesticide, i.e., thiram, and the limit of detection is as low as 1 × 10^–6^ mol/L (0.24 ppm). In addition, the good water adsorption performance of the paper-based SERS substrate makes it possible for direct detection of pesticide residues on the surface of fruit. The paper-based Au-Ag bimetallic SERS substrate developed in this study has better or comparable performance in detection limits compared to some recent studies in cellulose-based SERS substrates, showing a great potential in applications for rapid food safety detection.

## Data Availability

The original contributions presented in the study are included in the article/supplementary material, further inquiries can be directed to the corresponding authors.
